# Investigating Empathy-Like Responding to Conspecifics’ Distress in Pet Dogs

**DOI:** 10.1371/journal.pone.0152920

**Published:** 2016-04-28

**Authors:** Mylene Quervel-Chaumette, Viola Faerber, Tamás Faragó, Sarah Marshall-Pescini, Friederike Range

**Affiliations:** 1 Comparative Cognition, Messerli Research Institute, University of Veterinary Medicine, University of Vienna, 1 Veterinärplatz, Vienna, Austria; 2 MTA-ELTE Comparative Ethology Research Group, Pázmány Péter stny 1/C, H-1117 Budapest, Hungary; 3 Wolf Science Center, Dörfles 48A – 2115, Ernstbrunn, Austria; University of Sydney, AUSTRALIA

## Abstract

Empathy covers a wide range of phenomena varying according to the degree of cognitive complexity involved; ranging from emotional contagion, defined as the sharing of others’ emotional states, to sympathetic concern requiring animals to have an appraisal of the others’ situation and showing concern-like behaviors. While most studies have investigated how animals reacted in response to conspecifics’ distress, dogs so far have mainly been targeted to examine cross-species empathic responses. To investigate whether dogs would respond with empathy-like behavior also to conspecifics, we adopted a playback method using conspecifics’ vocalizations (whines) recorded during a distressful event as well as control sounds. Our subjects were first exposed to a playback phase where they were subjected either to a control sound, a familiar whine (from their familiar partner) or a stranger whine stimulus (from a stranger dog), and then a reunion phase where the familiar partner entered the room. When exposed to whines, dogs showed a higher behavioral alertness and exhibited more stress-related behaviors compared to when exposed to acoustically similar control sounds. Moreover, they demonstrated more comfort-offering behaviors toward their familiar partners following whine playbacks than after control stimuli. Furthermore, when looking at the first session, this comfort offering was biased towards the familiar partner when subjects were previously exposed to the familiar compared to the stranger whines. Finally, familiar whine stimuli tended to maintain higher cortisol levels while stranger whines did not. To our knowledge, these results are the first to suggest that dogs can experience and demonstrate “empathic-like” responses to conspecifics’ distress-calls.

## Introduction

Numerous studies have shown that animals display a sweep of emotions such as fear, anxiety, frustration, pain and pleasure [1,2,3]. Interestingly, while several studies have also demonstrated that some non-human primate [4–8] and non-primate species [9–11] are capable of recognizing others’ emotions, it is still an ongoing debate whether animals are also able to share others’ emotions and therefore experience a form of empathy. By some authors, empathy has been defined as the capacity to recognize, understand and share the others’ emotional state [12–17], while others additionally require the cognitive capacity to take the perspective of another while differentiating self from other [18–23]. According to one popular dissection of the term, the phenomena may be categorized based on the degree of cognitive complexity involved [22]. Based on this categorization, the lowest common denominator of all empathic processes is **emotional contagion** defined as being affected by another’s emotional state or sharing another’s emotion [22]. Emotional contagion does not necessarily involve self-awareness [19] and thus has been the most widely investigated empathetic response in non-human animals (see [22] for review). In contrast, s**ympathetic concern** and **empathetic perspective taking** are considered at the other end of the empathy spectrum, since they require individuals to have an appraisal of the others’ situation [22]. A common example of empathetic perspective taking is targeted helping, defined as help fine-tuned to another’s specific situation and goals. Empathetic perspective taking has been observed both in naturalistic and experimental contexts in primates, but also in non-primates species. For example, chimpanzees, have been shown to voluntary help others to get access to an out of reach desired item [24–26], elephants have been observed to show helping behavior towards ailing conspecifics [27] and rats placed in the presence of a trapped cage-mate spontaneously free their distressed companion [28]. Comforting behaviors directed at a distressed party are thought to represent an example of sympathetic concern and have been shown in chimpanzees [29], rooks [30] and dogs [31]. Overall, these empathic responses in animals have been found to be more likely to occur with kin and familiar partners [32,33].

Interestingly, while most studies have focused on demonstrating empathic-like responses between conspecifics (chimpanzees: [24,25,26], rooks: [30], elephants: [27], rats: [28,34,35]), the domestic dog has been targeted to investigate empathic responses to humans [36,37,38]. Due to the process of domestication, dogs have been suggested to have a heightened capacity to develop strong social-bonds with humans [39] and a good understanding of human communication [40,41], which might have enhanced their propensity to recognize and be sensitive to human’s emotions. Indeed it has been shown that dogs are capable of discriminating between human’s facial expressions of negative or positive emotions [11,41]. Furthermore, dogs have been suggested to show sympathetic concern towards a distressed human [36]. More precisely, in the latter study, dogs showed more comfort-offering behavior, such as approaching and touching, to a human when she was crying, compared to when the human was humming or talking [36]. Moreover, similarly to humans, it has been found that dogs’ cortisol levels increased when they were exposed to infant crying but not when exposed to infant babbling and white noise [37], which is in line with the definition of emotional contagion.

In contrast to the growing number of studies investigating dogs’ responses to human emotional expressions, to our knowledge there are no studies directly investigating dogs’ capacity for emotional recognition and empathic responses to conspecifics’ distress. Investigating this aspect will increase our understanding as to whether the responses are human specific e.g. brought about by specific selective forces during domestication, or are more generally derived from dogs’ capacity for empathic responding towards conspecifics. Indeed, free-ranging dogs live in social groups, coordinate the defense of their territory [42], and similarly to their closest living relatives—wolves -, exhibit a rich visual and vocal communicative system with their group members [43,44]. All these factors point to the potential need for synchronized action and hence a direct benefit from an ability to recognize and react to their conspecifics’ expression of emotion, or even share their inner state.

A recent study on pet dogs showed that the presence of behavioral mimicry (i.e. two dogs performing the same action simultaneously) during play was more likely to occur amongst closely bonded individuals and was found to increase the duration of the play sequence between the two individuals involved [38]. Since the observed behavioral mimicry was more likely to occur in response to behaviors signalling a positive emotional state, the authors suggested that it reflects a synchronization of the underlying emotions and is therefore likely to be a form of emotional contagion. Another potentially relevant study suggesting dogs’ capacity to recognize and be affected by a conspecifics’ expression of emotions, was conducted by Siniscalchi and colleagues [45] who showed that pet dogs demonstrated a higher cardiac activity and more stress-related behaviors (such as “tail-down, flattened ears, head low”) after being exposed to video-clips of conspecifics expressing left rather than right-biased tail wagging. Left-tail wagging has been shown to occur in response to negative stimuli, such as seeing a dominant unfamiliar dog, whilst, right-tail wagging is typically elicited by positive ones, such as seeing a dog’s owner [45]. Although authors did not link their results to emotional contagion, considering that subject’s physiological state was in line with the behavioral response of the observed dog, these results fit the definition of emotional contagion.

Considering the paucity of studies investigating empathy-like responses between dogs as well as the prior success in eliciting dogs’ behavioral and physiological responses to human vocalizations of distress [36,37], we conducted a playback experiment using conspecific vocalizations (whines) recorded during a distressful event. Dog whines are short, cyclic, high pitched and tonal calls [44] commonly containing biphonations (occurrence of a secondary, whistle like, extreme high fundamental frequency [46]) and predominantly used in negative, stressful contexts. The presence of nonlinear phenomena [47] and some results in other Canid species suggest that whines are individually distinguishable (wolves: [48]; dholes [49]) making them the ideal stimuli for our study.

To test whether dogs’ responses were simply elicited by specific sound features or by the underlying emotional valence of the vocalization, we also conducted a control condition with artificially generated sounds similar to whines in the inter-call intervals, amplitude and fundamental frequency. We predicted that if the emotional valence rather than sound features drive the response, dogs’ arousal would be higher when hearing whines than control sounds. Furthermore, since it has been shown that empathic-like responses are modulated by familiarity [32,33], we presented dogs with distress vocalizations recorded either from a familiar conspecific partner or a stranger dog.

During a ‘playback phase’, the subject dog, separated from its familiar partner, was subjected to either a whine (familiar or stranger whine) or a control sound. To test whether subjects experienced emotional contagion, we analyzed their physiological (heart rate and cortisol levels before and after the playback [37]), and behavioral responses (alertness and stress-related behaviors) to the playback stimuli. We predicted that, if dogs recognize the emotional content of others’ whines and experience emotional contagion, both in terms of being affected by the others’ emotion and sharing their inner state, they would show i) higher alertness and stress-related behaviors and ii) an increase in the heart rate and cortisol levels, in response to whines compared to control sounds. Additionally, we conducted a ‘reunion phase’, where the familiar partner entered the subject’s room immediately after the end of the playback, to test whether subjects show any comforting behavior towards their partners that could indicate sympathetic concern. We predicted that they would show more concern-like behaviors (e.g. affiliative behaviors, proximity seeking behaviors) towards their partner after experiencing the whine stimuli than the control sounds. Moreover, if whines are individually recognizable and emotional contagion is affected by familiarity, we expect dogs to show a stronger behavioral and physiological response after hearing a familiar rather than a stranger’s whine. Furthermore, in the reunion phase we expect dogs to show more concern-like behaviors towards their partner after hearing the partner’s vs. the stranger’s whines.

## Materials and Methods

### Ethical statement

Ethical approval was obtained from the ‘Ethik und Tierschutzkommission’ of University of Veterinary Medicine, committee in accordance with GSP (Good Scientific Practice) guidelines and national legislation (Protocol number: Ref. 20/07/97/2014)

### Subjects

Sixteen dog pairs of various breeds participated in this study (see Table 1). Dogs that had lived together for at least a year in the same household were selected. Within a pair, one dog was randomly assigned to be the subject (8 females and 8 males), while the other was the familiar partner. Dog owners voluntarily participated in the study and were recruited by e-mail using the internal database of the Clever Dog Lab, a scientific lab of the Unit of Comparative Cognition at the Messerli Research Institute (University of Veterinary Medicine, Medical University of Vienna, University of Vienna) where scientists carry out studies assessing dog’s cognitive abilities. Dog owners can voluntary register at the Clever Dog Lab and be later contacted via email to participate in the different studies. No special preconditions need to be met for being included in the dog owner list. In the present study, dog owners living with two or more dogs per household could participate in the testing. In the current study, three dogs’ owners living with three dogs also participated. However, from each household, only one subject dog and one familiar partner were tested. In the current study, the familiar partner dogs also served as strangers for the other subjects.

**Table 1:** Subjects’ identity and partners’ breed and body size information.

Subjects	Sex	Breed	Age	Familiar partner breed	Familiar partners’ age	Familiar partners’ sex	Stranger dogs breed	Familiar and stranger body size
Lizzy	F	French Bulldog	7	German shepherd-mix	9	F	Mix-breed	Large
Bounty	F	Australian shepherd	9	Australian shepherd	5	F	Bernese Mountain dog	Large
Xena	F	Hunting dog-mix	7	Hunting dog-mix	10	M	Staffordshire Bullterrier	Large
Neela	F	Australian shepherd	7	Australian shepherd	3	F	Labrador Gordon Setter-Mix	Large
Funny	F	Dachshund-mix	5	Dachshund-mix	6	F	Mix-breed	Small
Nell	F	Chinese Crested	7	Chinese Crested	8	F	Shiba Inu	Small
Achuk	F	Chesapeake bay retriever	8	Chesapeake bay retriever	6	M	Labrador	Large
Jessy	F	German shepherd-mix	8	French bulldog	7	F	Mix-breed	Small
Yori	M	Mix-breed	7	Staffordshire Bullterrier	8	F	Hunting dog-mix	Large
Aiko	M	Australian shepherd	4	Bernese Mountain dog	4	F	German shepherd-Mix	Large
Toivo	M	Shiba Inu	6	Shiba Inu	3	F	Mix-breed	Small
Charlie	M	Dachshund	5	Dachshund-mix	4	F	Dachshund-mix	Small
Elrond	M	Chesapeake bay retriever	6	Chesapeake bay retriever	11	F	Staffordshire Bullterrier	Large
Gizmo	M	Mix-breed	9	Mixed-breed	7	F	Shiba Inu	Small
Charly	M	Dachshund-mix	6	Dachshund-mix	4	F	Dachshund-mix	Small
Cookie	M	Cocker spaniel	6	Labrador	8	M	Chesapeake bay retriever	Large

However, the whine of an extra 3 dogs, living alone in their household, were also recorded and used as stranger whines during the test.

### General experimental procedure

Each subject participated in three different conditions. In the two experimental conditions, a whine from either the familiar partner or a stranger was played back to the subject. In the control condition, a computer-generated sound was played back (for details see below). Each stimulus was presented once to each subject on different days. A break of at least two weeks was maintained in between conditions. The order in which the stimuli were presented was counterbalanced and randomly assigned across subjects.

During the **playback phase**, subjects were separated from their familiar partner. The sound stimulus was played back to the subject dogs for 30 seconds, during which its behaviors were video recorded. In a **reunion phase** starting immediately after the playback phase, the familiar partner dog entered the testing room through the speaker room and interactions between the two dogs were recorded for 30 seconds. Additionally, the subjects’ heart rate was monitored during the entire experiment and cortisol samples were collected before and after each playback condition.

### Whines recording procedure

To ensure that whines corresponded to an actual vocalization of distress, we recorded dogs’ whines while they were in a stressful situation. To do so, dogs were separated from their owner for three minutes. The recording took place in a testing room (600 cm x 722 cm, see S1 Fig) at the Clever Dog Lab, several weeks before running the playback experiment. The experimenter entered the room first and hid within an opaque enclosure. Once the experimenter was in position the owner entered the room and attached the dog to a leash placed one meter away from the microphone, and then left the dog alone in the room. For the following three minutes the experimenter recorded any vocalization emitted by the dog. After the three minutes the owner returned, left the room with his/her dog and once outside, was allowed to praise and pet the dog. For the familiar condition, we used the 16 whines of the familiar partner. For the stranger condition, we used the whine of a dog matching in size of the familiar partner (see Table 1). Controlling for this factor was deemed potentially important because although no research has been carried out on dog whines, it has been shown that the fundamental frequencies of dog growls are affected by the dog’s size [50,51].

Whines were recorded using a directional microphone (Sennheiser ME66, Sennheiser, Wedemark Wennebostel, Germany) connected to a portable recorder (Zoom H4n Handy recorder, Zoom Corporation, Kanda-surugadai, Chiyoda-ku, Tokyo 101–0062, Japan). Sounds were recorded in uncompressed PCM WAV format with 44.1 kHz sampling rate and 16 bit depth. Moreover, to minimize any undesirable echo effect, blankets were fixed to the walls of the room.

### Stimuli preparation

Every stimulus was 30 sec long, RMS normalized to -30dB, uncompressed mono WAV files prepared from the whine recordings or generated with a computer in the case of control sounds. The whine stimuli were prepared by extracting 30 seconds from the original recording containing as many whine bouts as possible and free of any background noise. If there was no such continuous part on the recording we created 30 second long sounds by cutting out and removing noisy or long silent parts keeping the normal range of between whine-bout or between whine interval lengths (Adobe Audition CC, Praat).

To create the artificial control sound, the primary and, in case of biphonic whines [46], also the secondary fundamental frequencies of the respective familiar and stranger whines assigned to each subject, were measured and short harmonic sounds (0,7 +/- 0,3 sec) were generated using Praat [52] with a fundamental frequency falling in between the partner and the stranger dogs' whines. Then these short sounds were semi-randomized and a 30 sec long sequence with similar inter-call intervals as in the stranger and familiar whines was created with Adobe Audition. The control sounds were also RMS normalized to -30dB. The fact that the fundamental frequency of the generated control sounds fell in between the range of the familiar and stranger whines [53,54] controlled for the possibility that dogs merely responded to the acoustical features rather than the emotional valence of the call.

### Playback experiment

#### Room set-up

**Fig 1:**
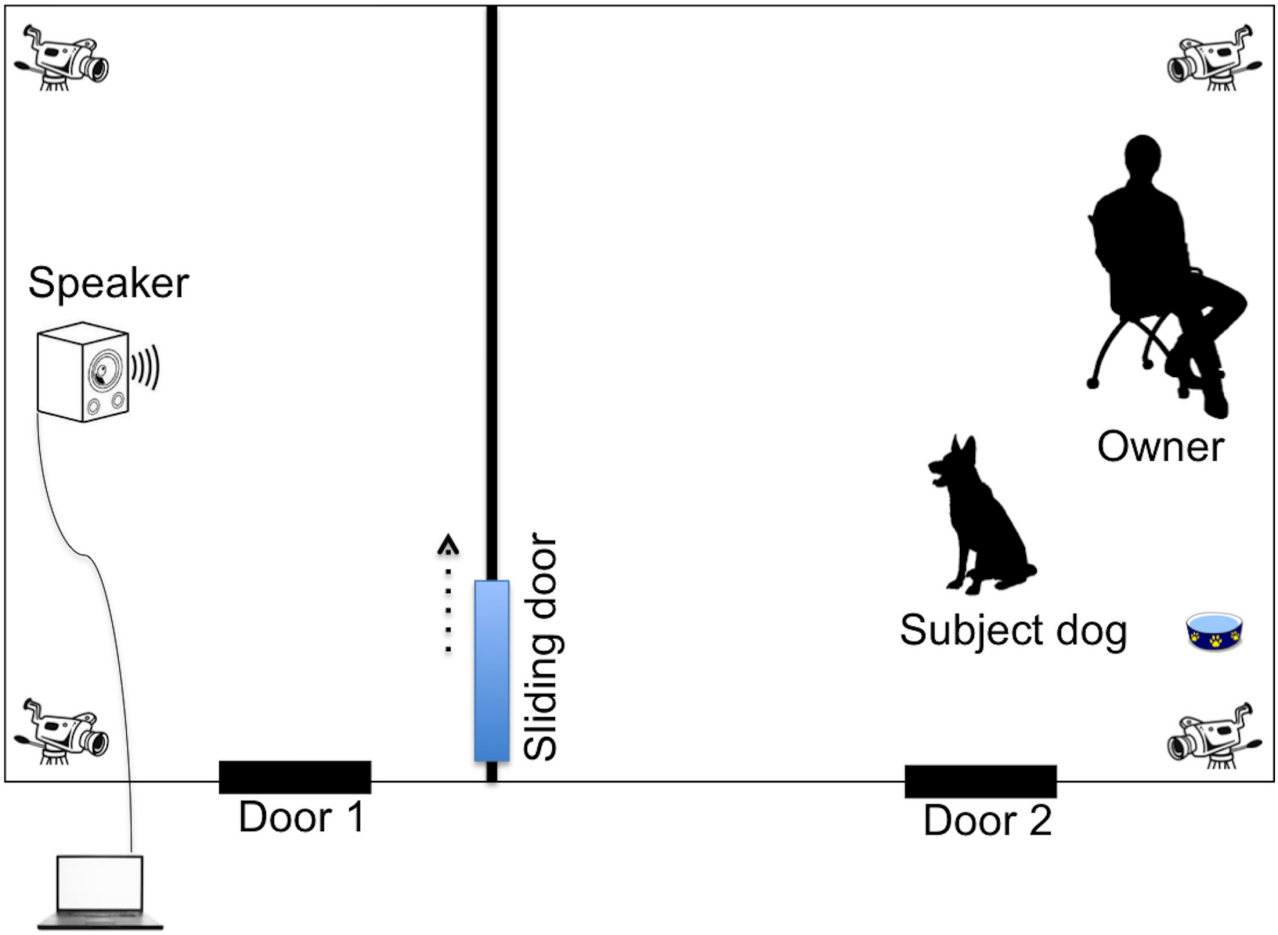
Set-up of the playback experiment.

The room was equipped with four wide angle cameras connected to a computer outside the testing room. This video system allowed the experimenter to monitor and record events inside the room. The room was divided into two areas by an opaque fence: i) the subject area (4,48 m x 6,00 m) and ii) the speaker area (2,6 x 6,00 m). Both areas were connected by a sliding door (0,75 x 1,1 m) in the fence (see Fig 1). A chair for the owner to sit on was placed in front of the wall in the subject area. A carpet and a water bowl were placed next to the chair.

On the other side of the opaque fence (speaker area), a loudspeaker (Yamaha HS 50M, Yamaha Corporation, Hamamatsu, Japan) was placed on the floor against the wall in the middle of the room (see Fig 1). The loudspeaker (volume-setting: 4 dB) was connected to a laptop (standard volume-setting, Asus Ultrabook UX32A) located outside the testing room from which the experimenter could play back the stimuli.

#### Testing procedure

The testing procedure included three different phases: 1) a thirty-minute waiting period, when both the owner and the subject dog stayed in the subject area of the testing room; 2) a 45 second playback phase immediately following the waiting period, when the sound stimuli was played back through the speaker; 3) a reunion phase, when the familiar partner dog entered the subject area 15 seconds after the end of the 30 second playback.

During the two first phases, an experimenter (E) stayed and interacted with the familiar partner dog in another room of the laboratory. This room was far enough from the testing room to prevent the subject and the partner from hearing what was happening in each other’s rooms.

1. During the first phase, both the owner and subject dog stayed in the subject area of the testing room for thirty minutes. The aim of this waiting phase was to familiarize the dogs with the testing room and allow them to become comfortable with their surroundings before taking the first saliva sample (pre-stimuli cortisol sample). During that time, the dog was free to explore the area while its owner was reading a book. At the end of the thirty minutes, E entered the room to collect the first saliva sample from the subject’s dog, corresponding to the pre-auditory stimulus sample.
2. Then the experimenter instructed the owner to put on a blindfold as well as headphones to prevent him/her from hearing the sound stimuli and unintentionally influencing the behavior of the subject dog. The experimenter left the room to play back the sound stimulus after approximately 2 minutes. The playback was thirty seconds long. In the meantime, E quickly picked up the familiar partner dog from its room.
3. Fifteen seconds after the end of the playback stimulus, E quietly entered the testing room with the familiar partner through the speaker area (door 1, see Fig 1). The experimenter immediately released the familiar partner dog into the subject’s area through the sliding door connecting the room’s two areas. As soon as the head of the familiar partner passed through the sliding door, the reunion phase started. E closed the sliding door behind the partner dog and left the testing room. For the following 30 seconds, both dogs could freely interact with each other. Then, the experimenter entered the room again to end the experiment. Consequently, the experimenter took a second saliva sample, corresponding to the post auditory stimulus sample, from the subject approximately 20 minutes after the end of the stimulus. Indeed, plasma and salivary cortisol levels are highly correlated in dogs [55] and a peak of salivary cortisol levels has been shown to occur around 18 minutes following an auditory stressor stimulus [56, 57]. For the saliva collection, the experimenter was holding a piece of cheese in front of the dog’s nose in order to activate the saliva flow, while gently rubbing the inside of the jaws with a cotton swap. This procedure is non-invasive and does not in itself cause salivary cortisol release in dogs [55]. The cotton part containing dogs’ saliva was cut off into a plastic tube (Microcentrifuge Tube, vol 1.5 ml) and stored at -20°C. The samples were then centrifuged for 10 minutes at 3750 revolutions per minute and cortisol levels were tested with enzyme immuno-assay (EIA).

Additionally, during the entire experiment, heart rate was monitored by using a Polar belt (RS800CX).

#### Behavior analyses

The video recordings of the playback and reunion phases were coded with Solomon Coder Beta 15.01.13 (Copyright András Péter, http://solomoncoder.com).

During the playback phase, the duration of gazing in the direction of the speaker, the time spent by the subjects in proximity of the opaque fence behind which the speaker was hidden (alertness behaviors), the duration of gazing to the owner, the time spent in proximity of the owner (Owner-orientated behaviors) as well as the duration of stress-related behaviors were measured (see Table 2 for definitions and categorization of behaviors). During the reunion phase, the time subjects spent in proximity to their familiar partners as well as the duration of affiliative behaviors initiated by the subject dogs toward their partners (see Table 2) were coded during the 30 seconds of the reunion phase.

**Table 2:** List and definitions of the coded behaviors.

Behavior variables	Definition
**PLAYBACK PHASE**	
Alertness behaviors	
Gaze at the speaker	The head of the dog is orientated towards the speaker hidden behind the center of the opaque fence.
Being in proximity of the opaque fence	The subject dog is located less than one body length from the opaque fence
Owner-orientated behaviors	
Gaze at the owner	The head of the subject is orientated towards the owner
Being in proximity of the owner	The subject is located less than one body length from the owner
Stress behaviors	
Stress-related behaviors	Lips licking, low body posture with the tail between the legs, yawning, scratching, shaking, whining.
**REUNION PHASE**	
Concern-like behaviors	
Duration of proximity to the partner	Time spent by the subject within one body length of its partner.
Affiliative behaviors directed at the partner	Time spent carrying out any of the following mutually exclusive affiliative behaviors directed at the partner: sniffing any body part of the partner, standing within one body length of partner whilst tail wagging, rubbing ones own body alongside that of the partner, play, and greeting (licking the lips of the partner, whilst tail wagging).

The videos were coded ‘blind’, in that the coders were not aware of the type of stimuli played back to the dogs. Inter observer reliability was tested on twenty percent of the videos by a second experimenter (all Cohen Kappa were above 0.86).

#### Physiological analyses

Considering dogs’ excitement when entering the lab, we measured the salivary cortisol variation by calculating the difference in cortisol level before and after each stimulus. For each dog, the mean heart rate during the playback phase for each condition was calculated. However, due to technical difficulties (abnormally high heart rate levels, artefacts and high rate of missing values) the data appeared potentially unreliable, hence the analyses pertaining to the heart rate measures are not reported.

#### Statistical analyses

Statistical analyses were calculated by using R studio (version 0.98.507 for mac) namely the function lmerTest of the R package lme4.

In order to investigate whether dogs were able to differentiate between whines and artificial control sounds, we conducted General Linear Mixed Models (LMM) with the subject’s identity as a random factor and the type of stimulus (whines versus artificial control sound) as well as the session number as fixed factors. As response factor terms we used: the mean duration of gazing to the speaker, the average time spent in proximity of the fence (log-transformed), the average duration of gazing to the owner (log-transformed), the duration of stress-related behaviors (reciprocal transformation), the average time subjects spent in proximity to the familiar partner (log-transformed), the mean duration of affiliative behaviors expressed by the subjects towards their partners, and the difference in the cortisol level before and after playback stimulus (inverse transformation).

Our second objective was to identify whether dogs could differentiate and would respond differently to whines according to the identity of the “caller”. Hence a second model was run comparing responses towards the familiar vs. stranger playback conditions.

We selected models of best fit based on the lowest Akaike’s information criterion (AIC) in a step-down process beginning with the two-way interaction of session and stimulus type among the predictors. Effect sizes of significant results are reported in the results section (see β–values).

## Results

a) Dogs’ reaction to the control and whine stimuli

During the playback phase, we found that the type of stimulus had an effect on both duration of dogs’ gaze towards the speaker (LMM: F_(1, 29)_ = 10.08, p = 0.003) and the time dogs spent in proximity of the opaque fence (LMM: F_(1, 31)_ = 5.476, p = 0.026,). Indeed, dogs spent more time gazing towards the speaker (LMM: β = 7.838, SE = 2.469, df = 29, p = 0.003) and staying in proximity of the opaque fence (LMM: β = 0.864, SE = 0.369, df = 31, p = 0.026) in the whine than control condition. Moreover, we found a main effect of session on the average duration of gaze towards the speaker (LMM: *F*_(2,29)_ = 6.42, *p* = 0.005) with subjects looking more towards the speaker in the last than in the two first sessions, regardless of the type of stimulus played back (LMM: session 1: β = -6.475, SE = 2.828, df = 29, t = -2.29, p = 0.029; session 2: β = -10.042, SE = 2.845, df = 29, t = -3.53, p = 0.001). This might be due to the fact that dogs, habituated to the set-up, were expecting to hear a sound and consequently were maybe more attentive to it in the last session. However, the amount of time dogs spent gazing at the owner (LMM: F_(1, 31)_ = 0.001, p = 0.97), staying in proximity of the owner (LMM: F_(1, 31)_ = 0.53, p = 0.47) as well as the variation in salivary cortisol level from before to after the playback (LMM: F_(1, 27)_ = 0.003, p = 0.95) did not differ between conditions.

Finally, dogs exhibited more stress-related behaviors when exposed to the conspecifics’ whines than when hearing the control stimuli (F_(1,29)_ = 5.247, p = 0.03; β = -0.20, SE = 0.08, df = 30, p = 0.02). However, the cortisol difference did not vary between conditions (LMM: F_(1, 27)_ = 0.003, p = 0.954).

During the reunion phase, whether the subjects heard a whine or a control sound affected the mean duration of affiliative behaviors initiated by the subjects towards their partner (LMM: F_(1, 31)_ = 7.685, p = 0.009) as well as the average time subjects spent in proximity of their partner (LMM: F_(1, 31)_ = 9.659, p = 0.004). Subjects spent more time in proximity of their partner (LMM: β = 0.319, SE = 0.10, df = 31, p = 0.004) and engaged in more affiliative behaviors (LMM: β = 2.762, SE = 0.99, df = 31, p = 0.009) in the whine than control conditions.

b) Dogs’ reaction to familiar vs. stranger whines

When looking at the duration of affiliative behaviors during the reunion phase, we found an interaction between session and condition (LMM: F_(1, 17)_ = 6.317, p = 0.022). Accordingly, we analyzed each session separately. We found that in the first session, subjects that were exposed to familiar whines (n = 5) spent more time expressing affiliative behaviors towards their familiar partner in the reunion phase than dogs that have heard the stranger whines (n = 5, F_(2, 13)_ = 8.31, p = 0.005).

Moreover, we found a tendency for a higher reduction in subject’s cortisol levels after the stranger condition compared with the familiar condition (LMM: F_(1, 6.09)_ = 4.66, β = 0.20, SE = 0.09, p = 0.07) suggesting that while the subjects were somewhat stressed before the playback, hearing a familiar dog whine maintained the stress level, whereas hearing a stranger’s whine did not.

Finally, none of the other behavioral responses (e.g. proximity to the fence, gazing towards the speaker etc.) were influenced by familiarity during the playback phase (LMMs: all p-value > 0.1, see S1 Table).

## Discussion

The aim of the present study was to test whether dogs show emotional contagion and sympathetic concern in response to distress vocalizations by conspecifics. First, we found a higher behavioral alertness (higher duration of gazing towards the speaker and time spent close to the opaque fence), more stress-related behaviors and an increase in concern-like behaviors (longer time spent in proximity and expressing affiliative behavior) in the whine compared with the control conditions, suggesting that dogs showed a form of emotional contagion and some sympathetic concern towards their partners. Moreover, dogs expressed more concern-like behaviors towards their familiar partner after hearing familiar whines compared with after stranger whines. In support of the latter, we also found that while familiar whine stimuli tended to maintain dog’s cortisol concentration at a higher level, stranger whines did not.

In their study, Yong and colleagues (2014) found that dogs’ arousal was higher when listening to infant crying in comparison to infant babbling or white noise [37]. More precisely, while alertness behaviors increased when dogs were listening to infant crying and white noise, the cortisol level only increased when dogs were exposed to infant crying. Since no differences in the duration per utterance, minimum pitch, fundamental frequency, or the first three formants emerged when comparing infant cries to babble, the authors concluded that dogs experienced emotional contagion when exposed to infant cries. Similarly, in the present study, dogs also showed more alertness behaviors in response to conspecifics’ whines than to control sounds excluding the possibility that dogs merely responded to the acoustical features rather than the emotional valence of the call. However, in contrast to the previous study, we found no increase in their salivary cortisol levels. Nonetheless, our subjects expressed more stress-related behaviors in response to the whine stimuli than to the control sounds suggesting that dogs experienced emotional contagion both in terms of *being affected* by the expression of others emotions and in terms of *sharing/converging* with others’ emotions. Furthermore, the fact that in the reunion phase, subjects demonstrated more concern-like behaviors towards their partners after being exposed to dog whines than the control stimuli supports the idea that dogs both perceived the negative emotional content of the whines and were affected by it.

In regard to the lack of physiological results there are two potential explanations: first, it is possible that the single acoustic modality of the stimuli rendered the signal too weak to provoke any measurable physiological variable of stress. This is unlikely since Yong and Ruffman (2014) showed that an auditory stimulus (human infant vocalizations) elicited an increase in dogs’ salivary cortisol levels [37]. However, the duration of the playback in the aforementioned study was much longer than the one used in the current experiment. It is therefore possible that our stimuli were too short to elicit dogs’ cortisol secretion. Secondly, Yong and Ruffman (2014) collected the pre-stimuli saliva sample of their subjects 40 minutes after they entered the lab, while in our set-up this baseline was taken after 30 minutes. Dogs’ excitement when entering the lab was quite high and could have resulted in an uncommonly high level of cortisol, providing a suboptimal baseline for comparison. In support of this latter possibility is the fact that, when comparing dogs’ reactions to stranger versus familiar whines, we did find a tendency for a difference in cortisol levels between the two whine conditions. It seems that cortisol levels when first arriving at the lab gradually decreased over the course of the test when stranger whines were played, but remained as high when the familiar whines were heard instead. Interestingly, this latter result is in line with the findings that familiarity enhances emotional contagion [18,33,58,59]. Additionally, in our reunion phase, dogs demonstrated more concern-like behavior towards their partners after being exposed to their whines compared with when they heard the stranger whines. Unfortunately, this effect was limited to only the first session, which is likely due to a habituation effect with subjects ceasing to react towards their partner after further testing.

Additionally, some could argue that dogs experiencing the stranger whine might have been surprised to see their familiar partner entering the room and therefore showed less reaction towards them. However, in cognitive studies using a violation of expectancy paradigm, dogs showed a higher response in the “surprise” condition than in the non-surprised one [60,61], i.e. the opposite result to what was found in our experiment. Indeed, our familiar condition, where there was no surprise effect, was the condition in which dogs actually demonstrated a stronger reaction (i.e. more affiliative behavior) towards their partner.

Nonetheless, it is important to keep in mind that the difference observed in response to the two types of whines (familiar versus stranger whines) was based on a small sample size (5 individuals in each whine conditions). Consequently, results relating to this comparison need to be interpreted with caution.

The concern-like behaviors towards the partner, occurring both more often following distress vocalizations of conspecifics compared with control sounds and more so after familiar compared with stranger whines, at least in the first session, are comparable to consolation or comfort-offering behaviors (sympathetic-concern). These behaviors are known to usually be exhibited after agonistic situations and believed to have a calming effect on the receiver [29,31,62]. It is also in line with findings by Custance & Mayer (2012) who found greater comfort-offering (i.e. friendly approaches and gazing) occurring towards a distress-showing crying person compared to a talking or humming one [36]. In our experiment, dogs’ concern-like behaviors towards the partner might have been triggered by the subjects’ desire for social contact in order to diminish their own distress rather than comforting their supposedly distressed familiar partners. However, as Custance & Mayer (2012) already suggested for their study, if dogs were egoistically motivated, we would expect them to go to the non-distressed individual present in the room (the owner) to seek comfort. But such a reaction did not emerge. Nonetheless, we cannot completely exclude that the fact that the owners had their backs turned and were wearing a blindfold might have discouraged our subjects from seeking comfort from them.

Taken together, dogs’ reaction to conspecifics’ distress in the current study is in line with the definition of emotional contagion and sympathetic concern proposed by de Waal [22]. However, this study has some limitations. First, one could argue that dogs might have simply reacted with increased stress-related behaviors to a dog vs. non-dog sound without necessarily perceiving the emotional content underlying the vocalization. A ‘neutral’ dog or even a ‘positive’ dog vocalization would have controlled for this aspect. One difficulty here is the choice and successful elicitation of neutral/positive dog vocalizations, since positive valence vocalizations are not as frequent in dogs [44]. Moreover, there is a serious risk of habituation effects occurring when increasing the number of stimuli dogs are exposed to. Second, we could not exclude that the relationship between our subjects and their familiar partners influenced our results—e.g. that dogs with “bad” relationships may have reacted in a less concerned manner than those with a closer bond. In a similar direction, subjects living with familiar partners who whine often may have become habituated to such a response showing little or no reaction to it. These two aspects might explain the high variability in dogs’ response to the familiar whine and need to be tested in the future.

## Conclusions

To sum up, when exposed to whines, dogs showed more alertness behaviors compared to when exposed to acoustically similar control sounds. They also demonstrated more comfort-offering behaviors towards their familiar partners following whines than after the control stimuli. Moreover, when looking only at the first session, this comfort-offering was biased towards the familiar partner when subjects were previously exposed to the familiar whine compared to the stranger one. Finally, familiar whine stimuli tended to maintain dog’s cortisol levels higher while stranger whines did not. These results suggest that dogs experience and demonstrate “empathic-like” responses to conspecific distress-calls.

## Supporting Information

S1 Data Supporting data. (XLSX)

S1 Fig A) Bird’s-eye view drawing of the recording room, B) Side view picture of the recording room.
(DOCX)

S1 Table Anova output of the best-fit models after reduction. For all models the only predictor that remained after reduction was the type of whine (familiar vs stranger).
(DOCX)
