# Supplementary figures and images for: Investigating Empathy-Like Responding to Conspecifics’ Distress in Pet Dogs
Source: PLoS One. 2016 Apr 28;11(4):e0152920. doi: 10.1371/journal.pone.0152920 (PMC4849795; doi:10.1371/journal.pone.0152920)

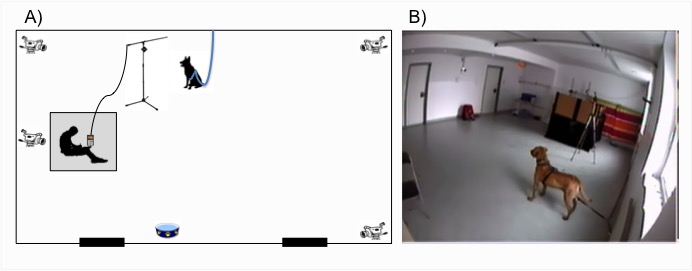


Figure S1. A) Upper view drawing of the recording room, B) Side view picture of the recording room.

Supplement: S1 Fig — A) Bird’s-eye view drawing of the recording room, B) Side view picture of the recording room. (DOCX) [file pone.0152920.s002.docx]
