# Supplementary material for: Investigating Empathy-Like Responding to Conspecifics’ Distress in Pet Dogs
Source: PLoS One. 2016 Apr 28;11(4):e0152920. doi: 10.1371/journal.pone.0152920 (PMC4849795; doi:10.1371/journal.pone.0152920)
Supplement: S1 Table — For all models the only predictor that remained after reduction was the type of whine (familiar vs stranger). (DOCX) [file pone.0152920.s003.docx]

Table. S1. Anova output of the best-fit models after reduction. For all models the only predictor that remained after reduction was the type of whine (familiar vs stranger).

| Response variables | F-values | p-values |
| --- | --- | --- |
| Duration of gazing at the speaker | F_(1, 15)_ = 0.81 | p = 0.38 |
| Duration of proximity fence | F_(1, 15)_ = 0.52 | p = 0.48 |
| Duration stress behaviors | F_(1, 15)_ = 2.95 | p = 0.11 |
| Duration of gazing at the owner | F_(1, 15)_ = 0.45 | p = 0.51 |
| Duration of proximity owner | F_(1, 15)_ = 2.38 | p = 0.14 |
